# Supplementary figures and images for: Genomic and phenotypic evolution of nematode-infecting microsporidia
Source: PLoS Pathog. 2023 Jul 20;19(7):e1011510. doi: 10.1371/journal.ppat.1011510 (PMC10393165; doi:10.1371/journal.ppat.1011510)

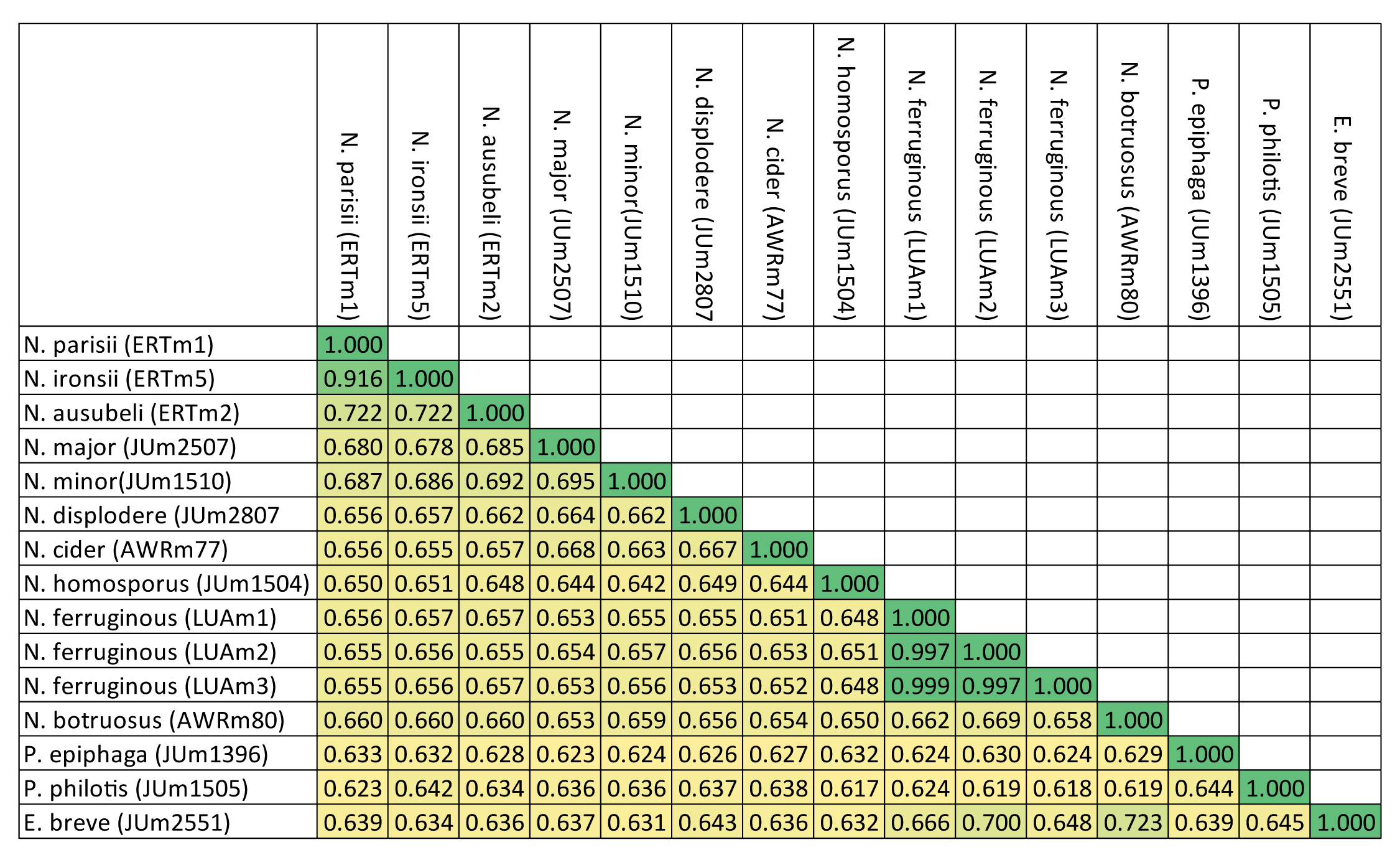

Supplement: S1 Fig — The pairwise nucleotide identity of the three N. ferruginous assemblies and the other nematode-infecting microsporidia genomes were calculated and displayed as a heat map. (TIF) [file ppat.1011510.s001.tif]

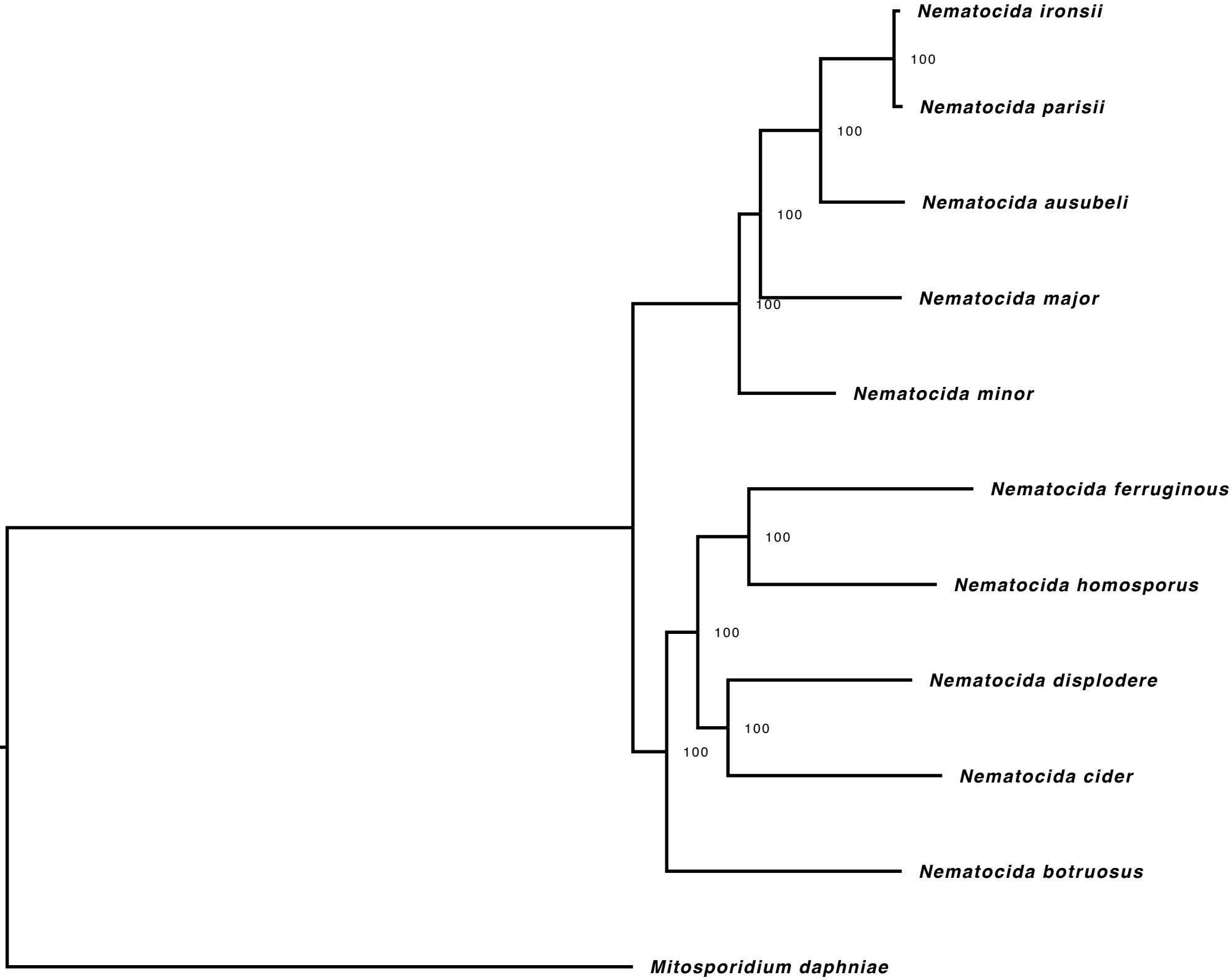

0.2

Supplement: S2 Fig — The phylogeny of 10 Nematocida species was determined from single-copy orthologs identified using OrthoMCL. Phylogenetic tree was generated using RaxML. M. daphniae is shown as an outgroup. Bootstrap values are indicated at each node. Scale indicates changes per site. (PDF) [file ppat.1011510.s002.pdf]

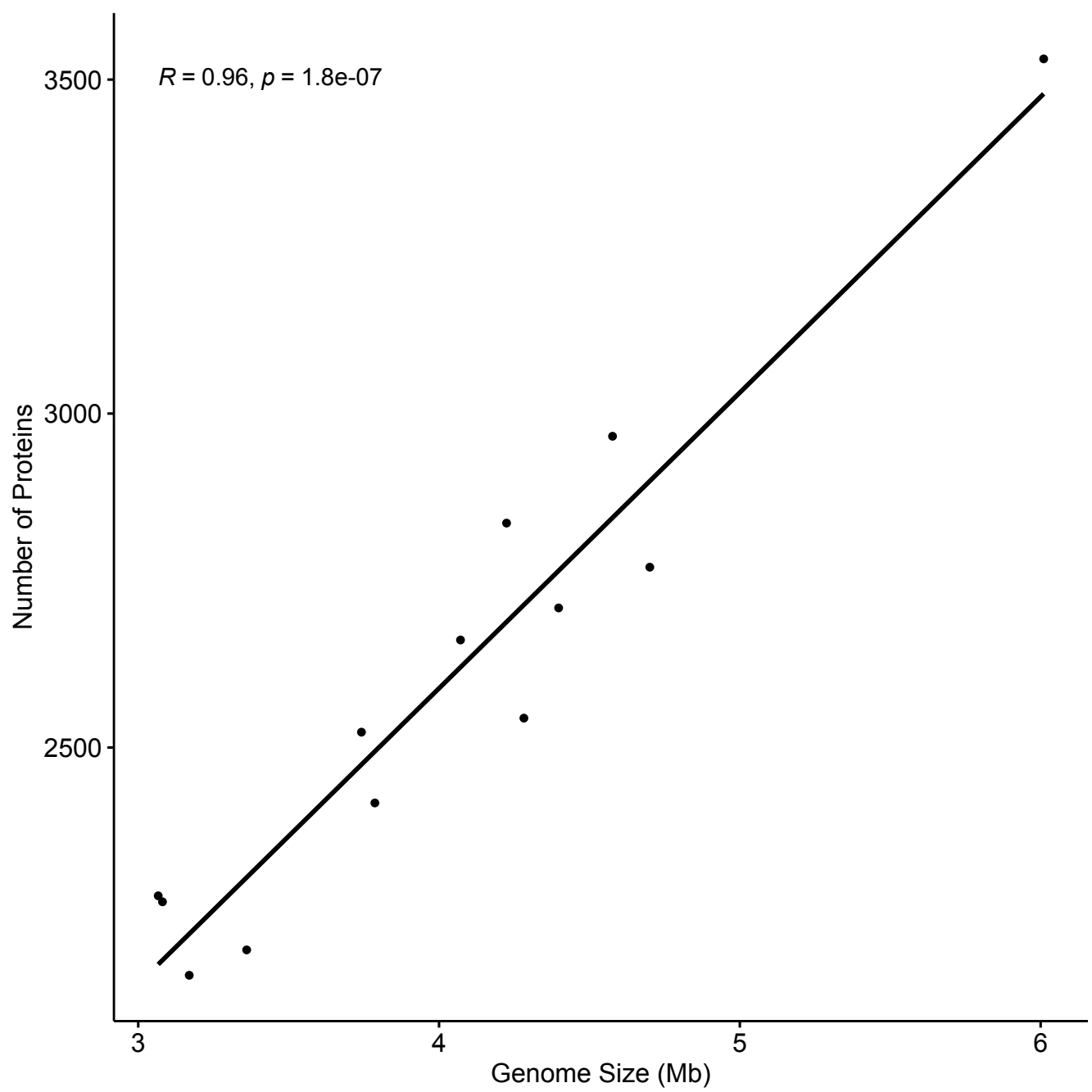

Supplement: S3 Fig — The correlation between genome size and protein number in 13 nematode-infecting microsporidia genomes is shown as a scatter plot. Pearson correlation coefficient and p-value are shown in the top left. (PDF) [file ppat.1011510.s003.pdf]

# GO:0005975 Carbohydrate metabolic process

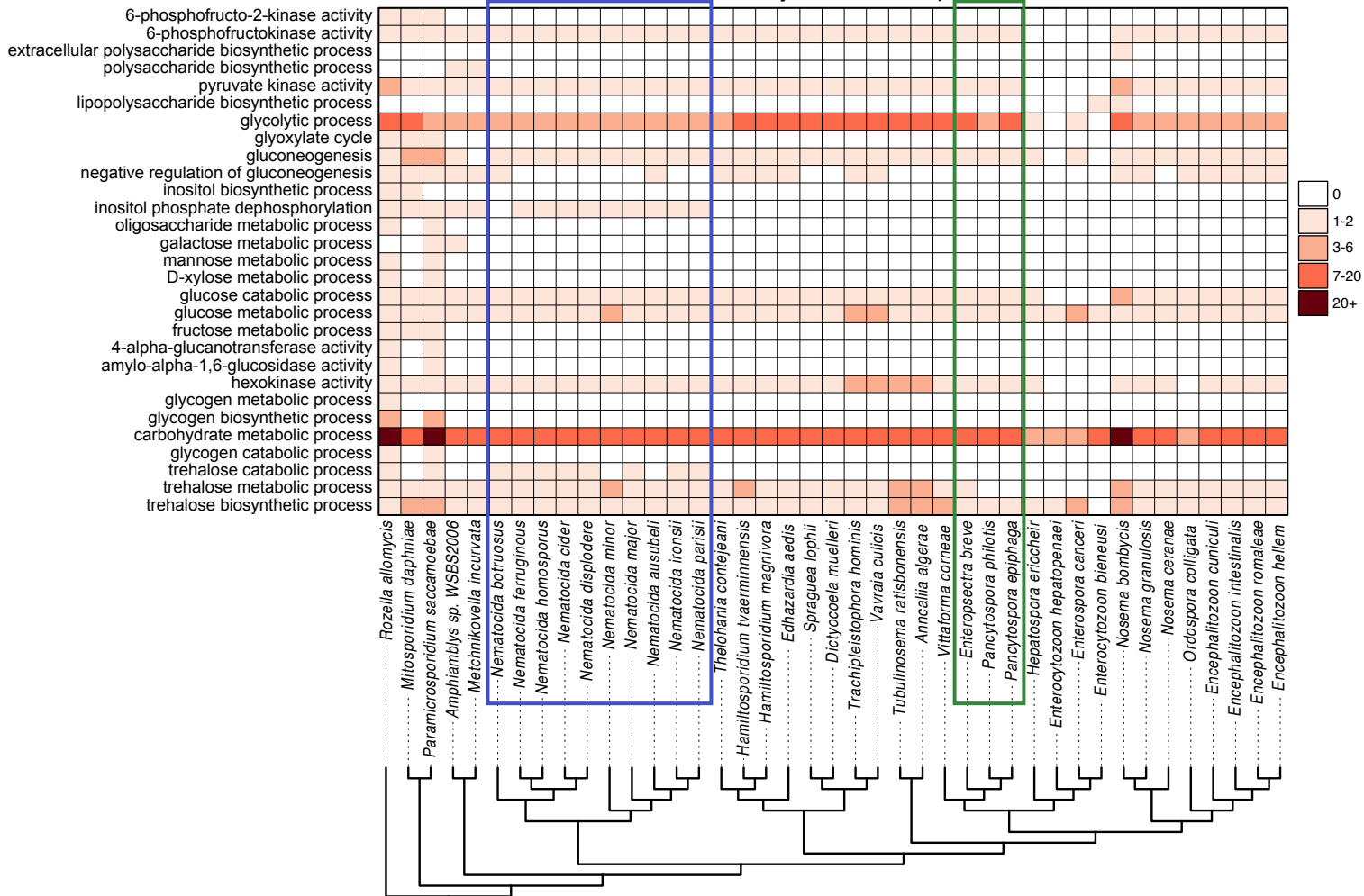

Supplement: S4 Fig — Membership of proteins from R. allomycis and 40 microsporidia species in descendant GO terms from the Pombe GO-slim category “carbohydrate metabolic process” was determined. The number of proteins from each species determined to belong to each Go term is shown as a heatmap with GO-slim categories in rows and microsporidia species in columns. Only descendant GO terms that contain at least one protein from any of these species is shown. Legend for the number of proteins in each cell is shown at the right. Phylogenetic tree, shown at bottom, was constructed using Orthofinder. Several species (Pseudoloma neurophilia, Dictyocoela roeselum, Cucumispora dikerogammari, and Nosema apis) were excluded due to poorer quality genome assemblies (See Fig 1). Nematocida species are highlighted with a blue box. Enteropsectra and Pancytospora species are highlighted with a green box. (PDF) [file ppat.1011510.s004.pdf]

# GO:0006091 Generation of precursor metabolites and energy

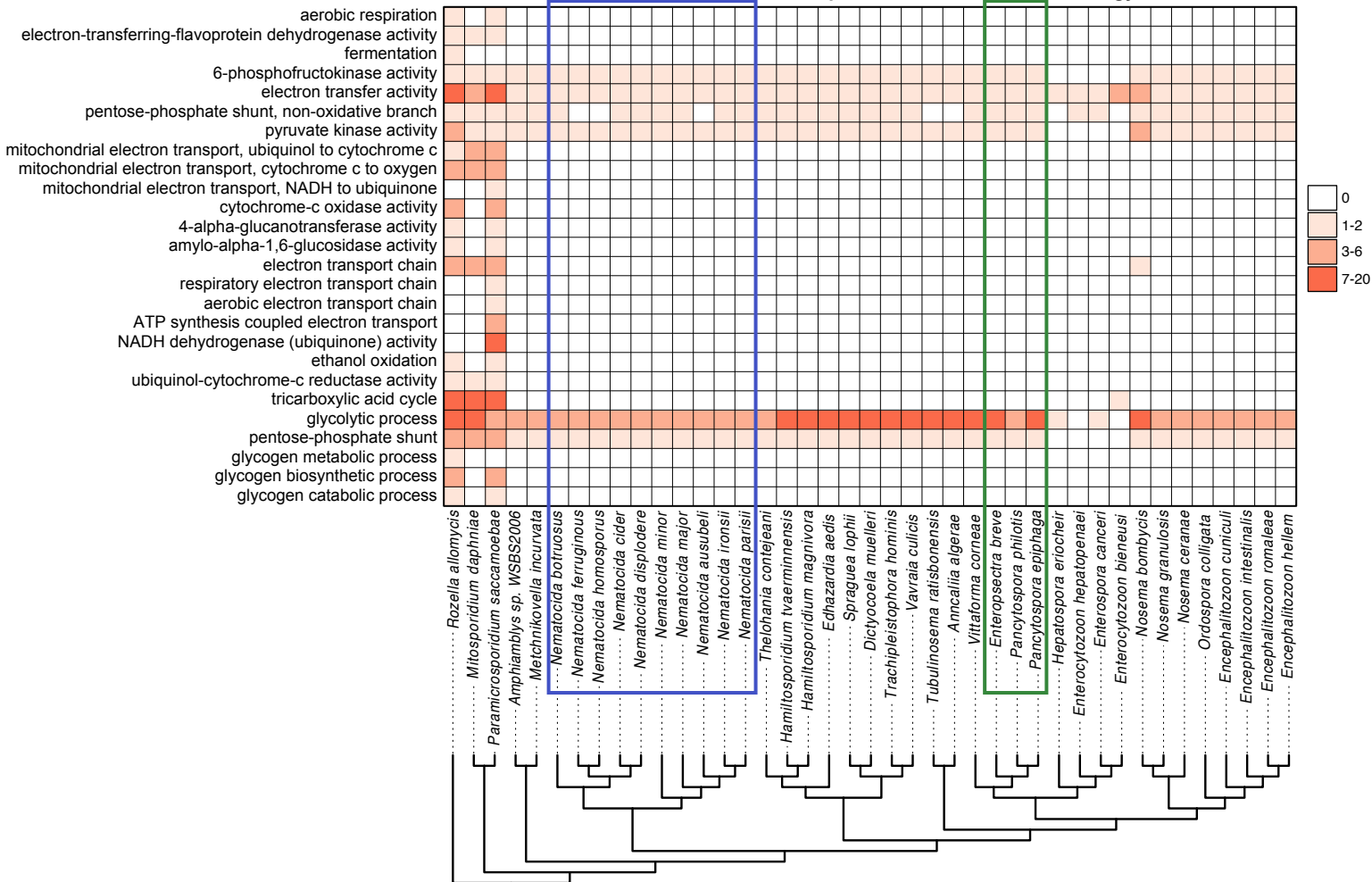

Supplement: S5 Fig — Membership of proteins from R. allomycis and 40 microsporidia species in descendant GO terms from the Pombe GO-slim category “generation of precursor metabolites and energy” was determined. The number of proteins from each species determined to belong to each Go term is shown as a heatmap with GO-slim categories in rows and microsporidia species in columns. Only descendant GO terms that contain at least one protein from any of these species is shown. Legend for the number of proteins in each cell is shown at the right. Phylogenetic tree, shown at bottom, was constructed using Orthofinder. Several species (Pseudoloma neurophilia, Dictyocoela roeselum, Cucumispora dikerogammari, and Nosema apis) were excluded due to poorer quality genome assemblies (See Fig 1). Nematocida species are highlighted with a blue box. Enteropsectra and Pancytospora species are highlighted with a green box. (PDF) [file ppat.1011510.s005.pdf]

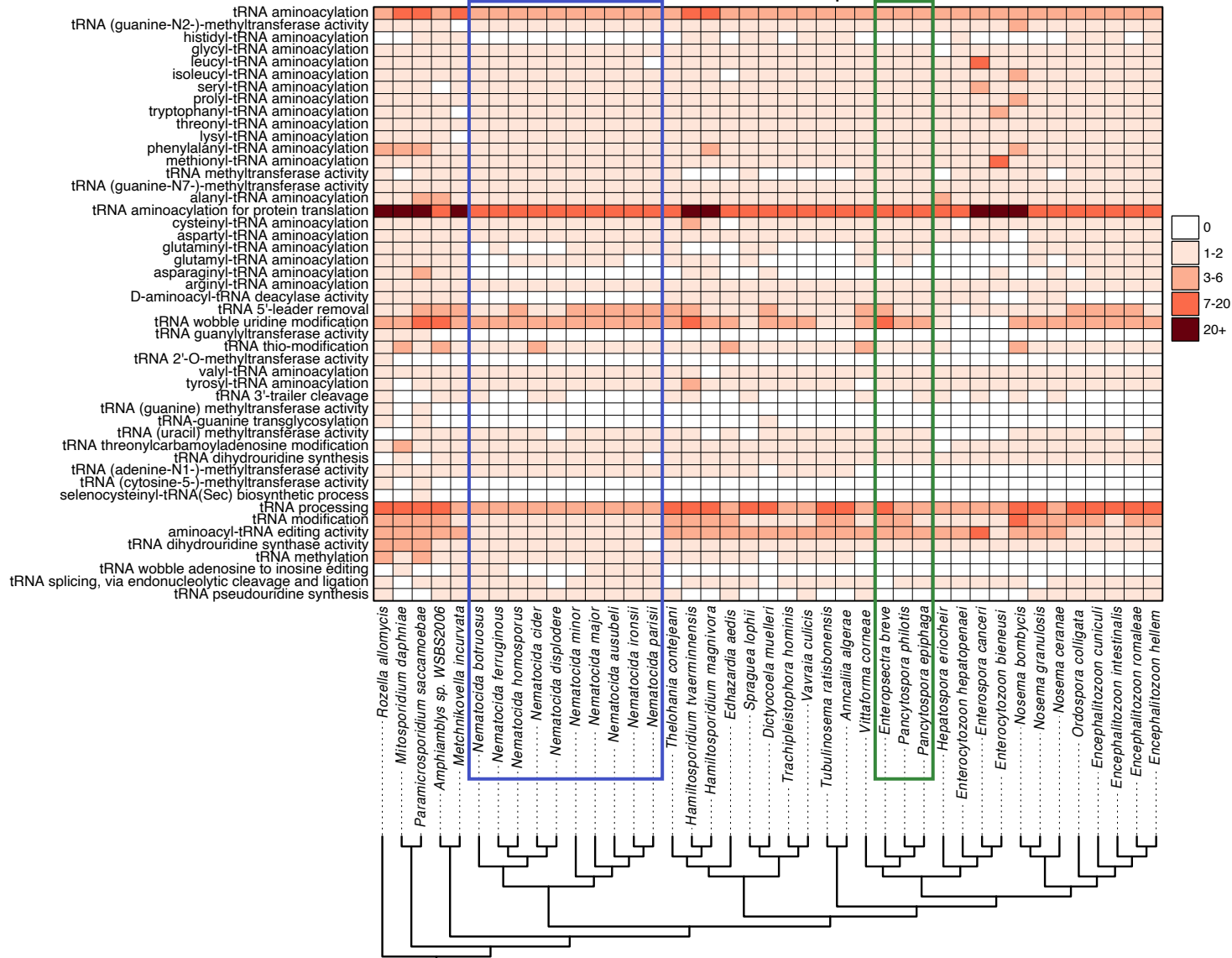

Supplement: S6 Fig — Membership of proteins from R. allomycis and 40 microsporidia species in descendant GO terms from the Pombe GO-slim category “tRNA metabolic process” was determined. The number of proteins from each species determined to belong to each Go term is shown as a heatmap with GO-slim categories in rows and microsporidia species in columns. Only descendant GO terms that contain at least one protein from any of these species is shown. Legend for the number of proteins in each cell is shown at the right. Phylogenetic tree, shown at bottom, was constructed using Orthofinder. Several species (Pseudoloma neurophilia, Dictyocoela roeselum, Cucumispora dikerogammari, and Nosema apis) were excluded due to poorer quality genome assemblies (See Fig 1). Nematocida species are highlighted with a blue box. Enteropsectra and Pancytospora species are highlighted with a green box. (PDF) [file ppat.1011510.s006.pdf]

## GO:0006520 Cellular amino acid metabolic process

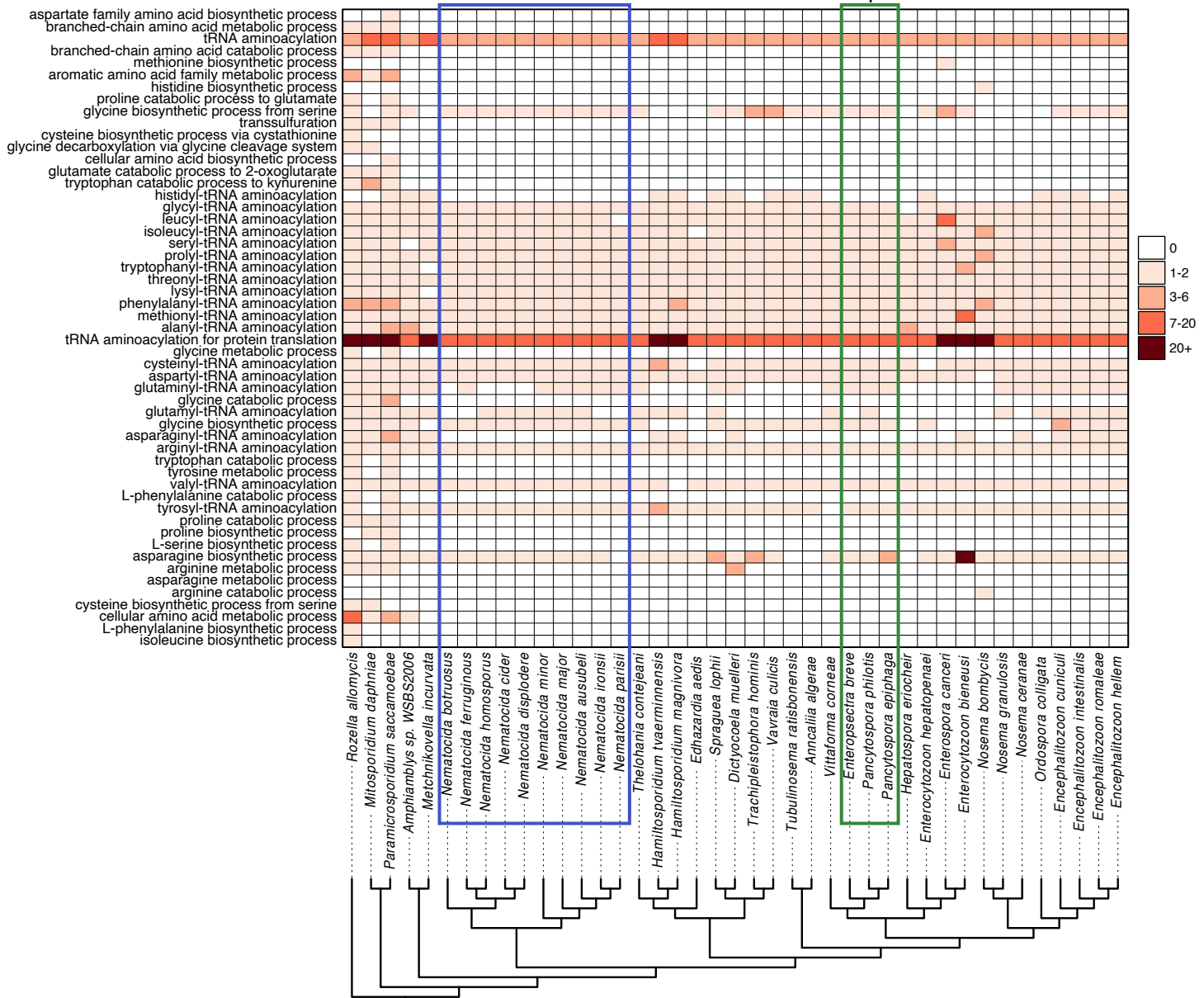

Supplement: S7 Fig — Membership of proteins from R. allomycis and 40 microsporidia species in descendant GO terms from the Pombe GO-slim category “cellular amino acid metabolic process” was determined. The number of proteins from each species determined to belong to each Go term is shown as a heatmap with GO-slim categories in rows and microsporidia species in columns. Only descendant GO terms that contain at least one protein from any of these species is shown. Legend for the number of proteins in each cell is shown at the right. Phylogenetic tree, shown at bottom, was constructed using Orthofinder. Several species (Pseudoloma neurophilia, Dictyocoela roeselum, Cucumispora dikerogammari, and Nosema apis) were excluded due to poorer quality genome assemblies (See Fig 1). Nematocida species are highlighted with a blue box. Enteropsectra and Pancytospora species are highlighted with a green box. (PDF) [file ppat.1011510.s007.pdf]

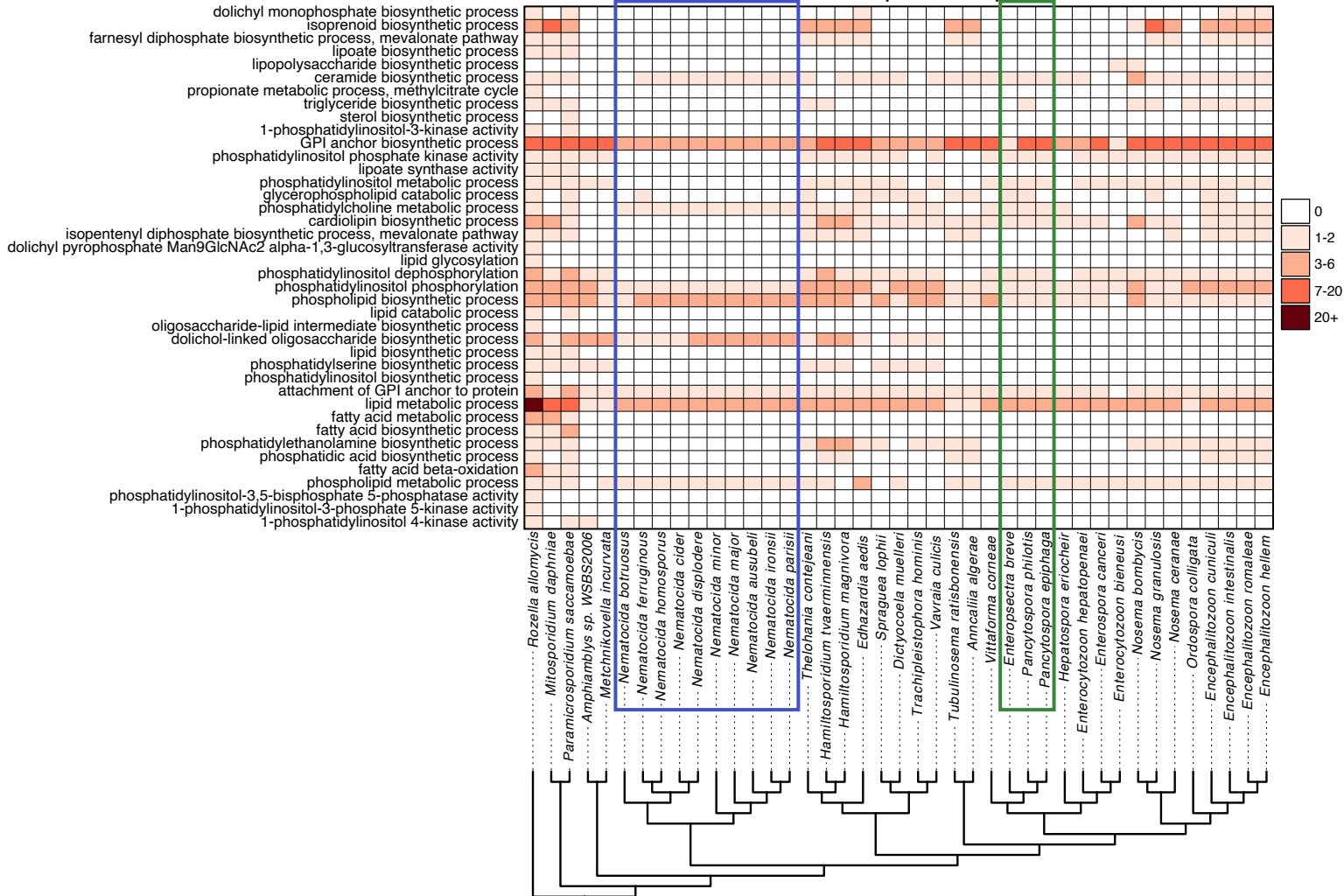

Supplement: S8 Fig — Membership of proteins from R. allomycis and 40 microsporidia species in descendant GO terms from the Pombe GO-slim category “lipid metabolic process” was determined. The number of proteins from each species determined to belong to each Go term is shown as a heatmap with GO-slim categories in rows and microsporidia species in columns. Only descendant GO terms that contain at least one protein from any of these species is shown. Legend for the number of proteins in each cell is shown at the right. Phylogenetic tree, shown at bottom, was constructed using Orthofinder. Several species (Pseudoloma neurophilia, Dictyocoela roeselum, Cucumispora dikerogammari, and Nosema apis) were excluded due to poorer quality genome assemblies (See Fig 1). Nematocida species are highlighted with a blue box. Enteropsectra and Pancytospora species are highlighted with a green box. (PDF) [file ppat.1011510.s008.pdf]

GO:0006790 Sulfur compound metabolic process

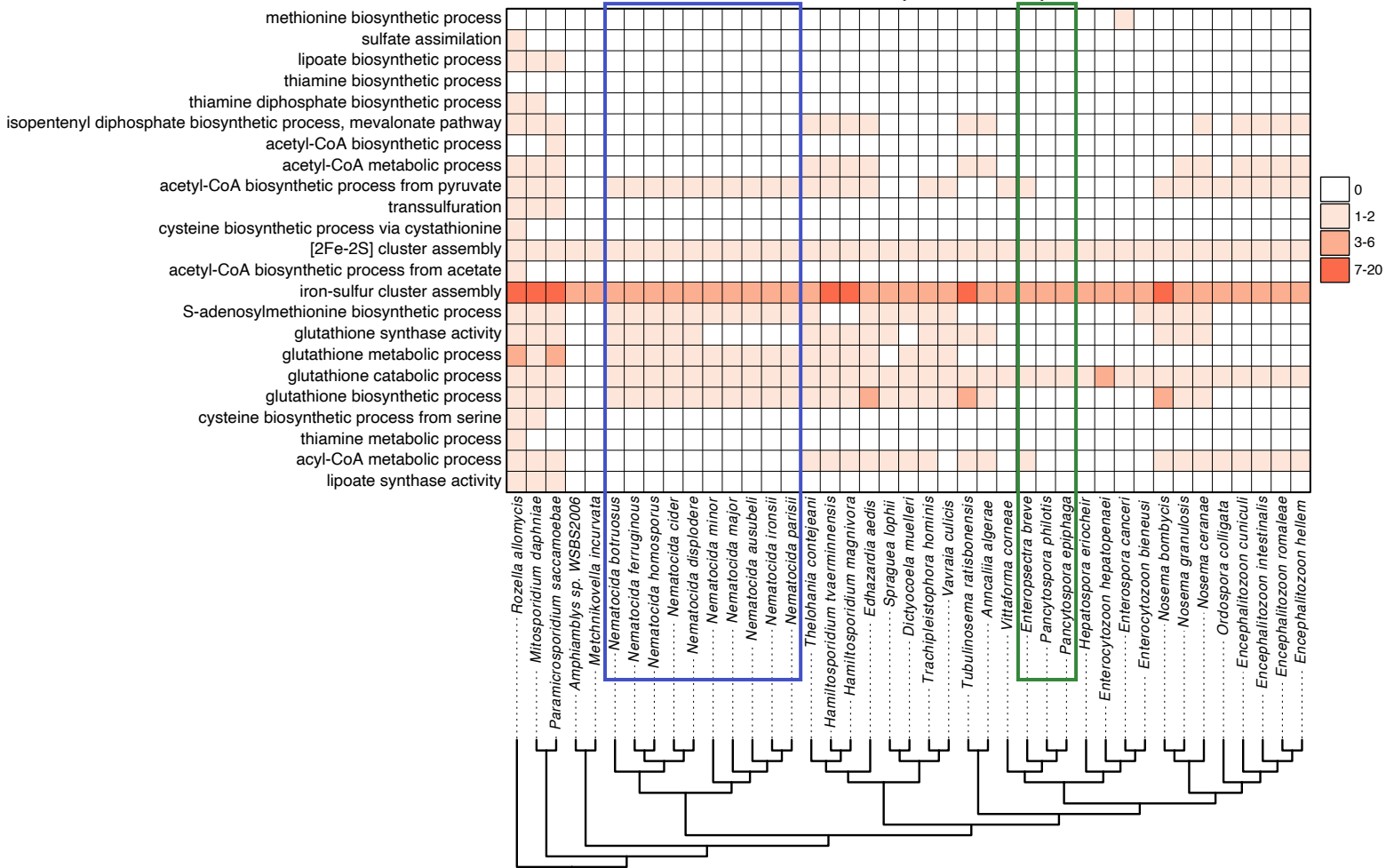

Supplement: S9 Fig — Membership of proteins from R. allomycis and 40 microsporidia species in descendant GO terms from the Pombe GO-slim category “sulfur compound metabolic process” was determined. The number of proteins from each species determined to belong to each Go term is shown as a heatmap with GO-slim categories in rows and microsporidia species in columns. Only descendant GO terms that contain at least one protein from any of these species is shown. Legend for the number of proteins in each cell is shown at the right. Phylogenetic tree, shown at bottom, was constructed using Orthofinder. Several species (Pseudoloma neurophilia, Dictyocoela roeselum, Cucumispora dikerogammari, and Nosema apis) were excluded due to poorer quality genome assemblies (See Fig 1). Nematocida species are highlighted with a blue box. Enteropsectra and Pancytospora species are highlighted with a green box. (PDF) [file ppat.1011510.s009.pdf]

## GO:0016071 mRNA metabolic process

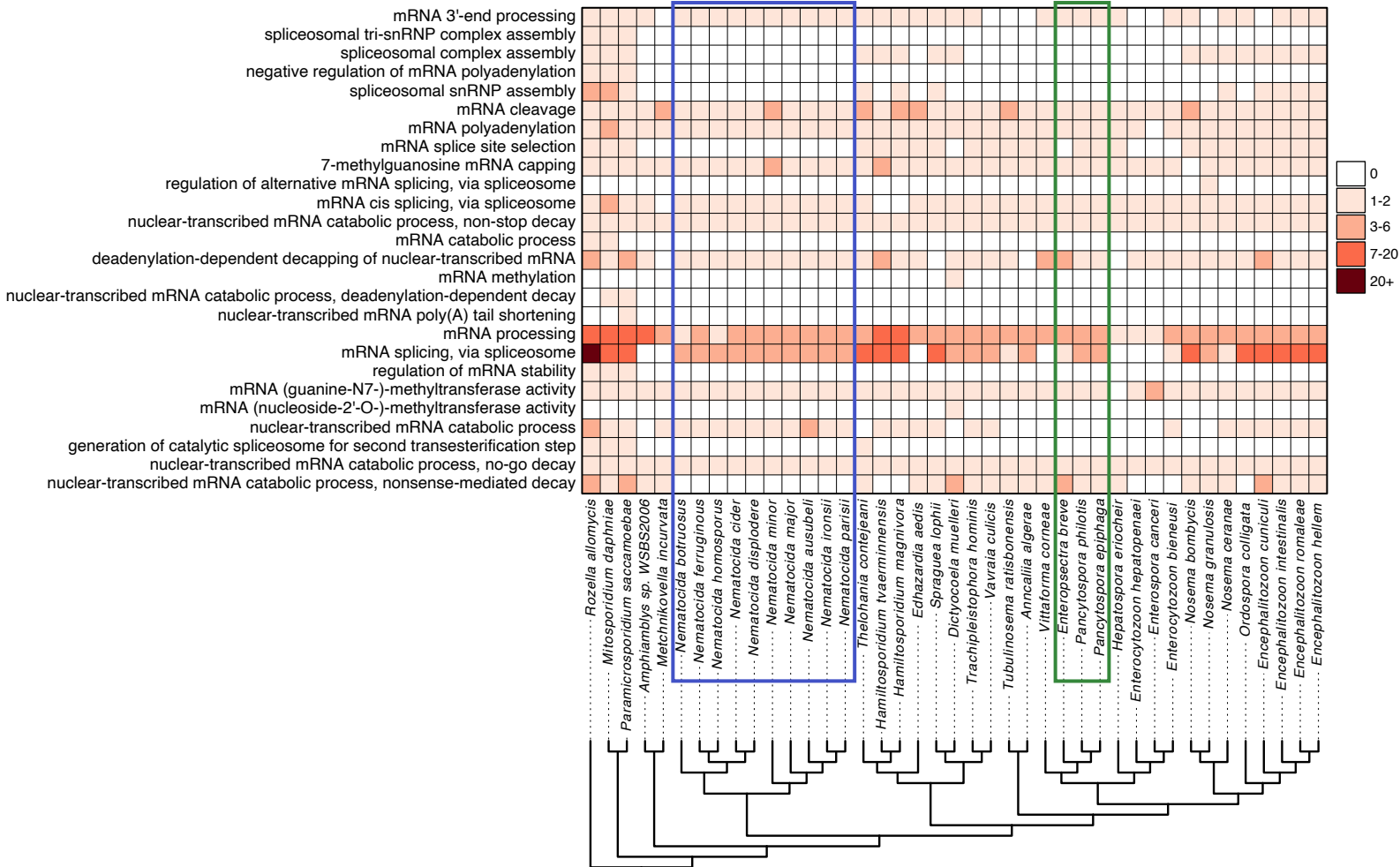

Supplement: S10 Fig — Membership of proteins from R. allomycis and 40 microsporidia species in descendant GO terms from the Pombe GO-slim category “mRNA metabolic process” was determined. The number of proteins from each species determined to belong to each Go term is shown as a heatmap with GO-slim categories in rows and microsporidia species in columns. Only descendant GO terms that contain at least one protein from any of these species is shown. Legend for the number of proteins in each cell is shown at the right. Phylogenetic tree, shown at bottom, was constructed using Orthofinder. Several species (Pseudoloma neurophilia, Dictyocoela roeselum, Cucumispora dikerogammari, and Nosema apis) were excluded due to poorer quality genome assemblies (See Fig 1). Nematocida species are highlighted with a blue box. Enteropsectra and Pancytospora species are highlighted with a green box. (PDF) [file ppat.1011510.s010.pdf]

GO:0055086 nucleobase-containing small molecule metabolic process

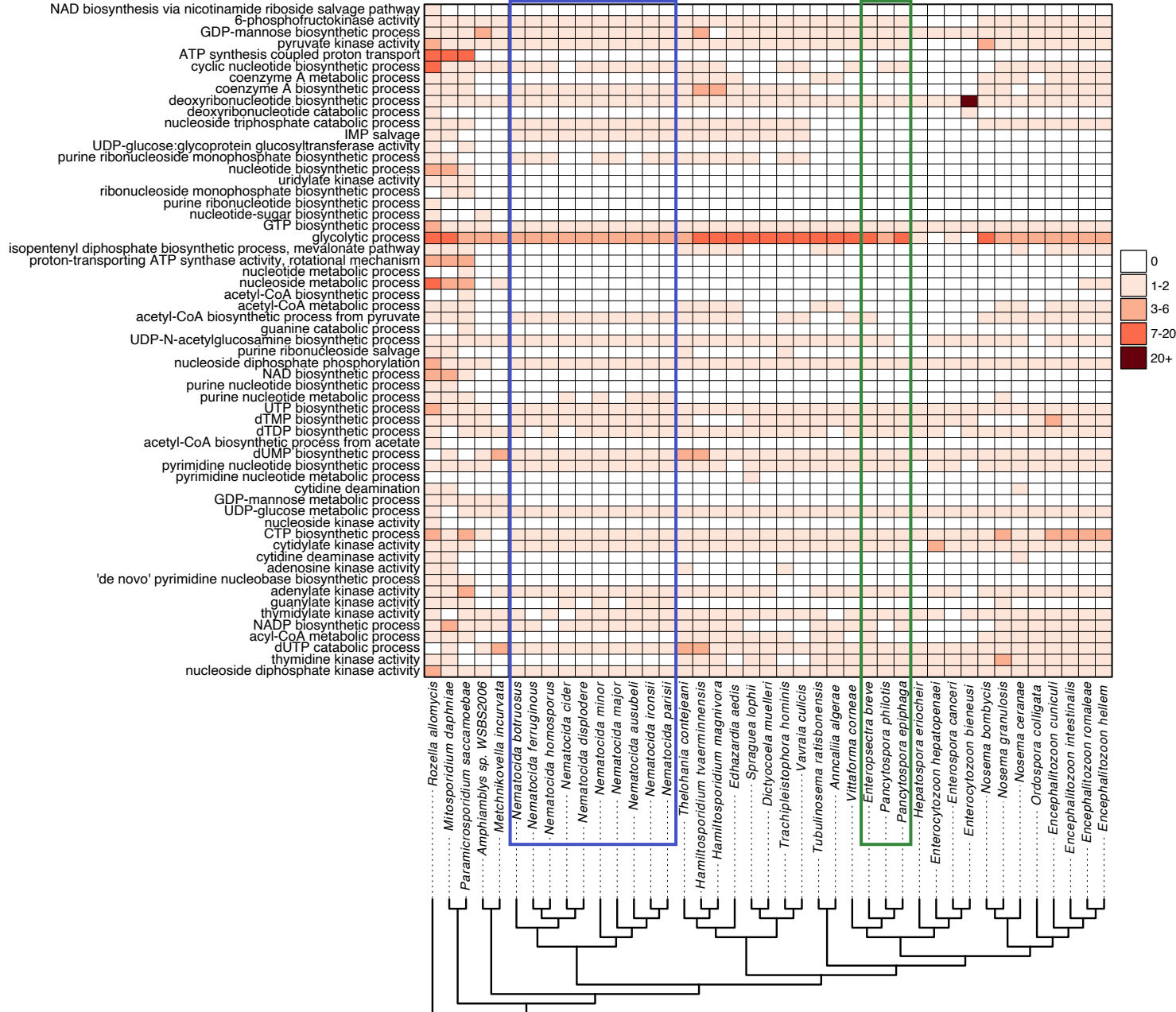

Supplement: S11 Fig — Membership of proteins from R. allomycis and 40 microsporidia species in descendant GO terms from the Pombe GO-slim category “nucleobase-containing small molecule metabolic process” was determined. The number of proteins from each species determined to belong to each Go term is shown as a heatmap with GO-slim categories in rows and microsporidia species in columns. Only descendant GO terms that contain at least one protein from any of these species is shown. Legend for the number of proteins in each cell is shown at the right. Phylogenetic tree, shown at bottom, was constructed using Orthofinder. Several species (Pseudoloma neurophilia, Dictyocoela roeselum, Cucumispora dikerogammari, and Nosema apis) were excluded due to poorer quality genome assemblies (See Fig 1). Nematocida species are highlighted with a blue box. Enteropsectra and Pancytospora species are highlighted with a green box. (PDF) [file ppat.1011510.s011.pdf]

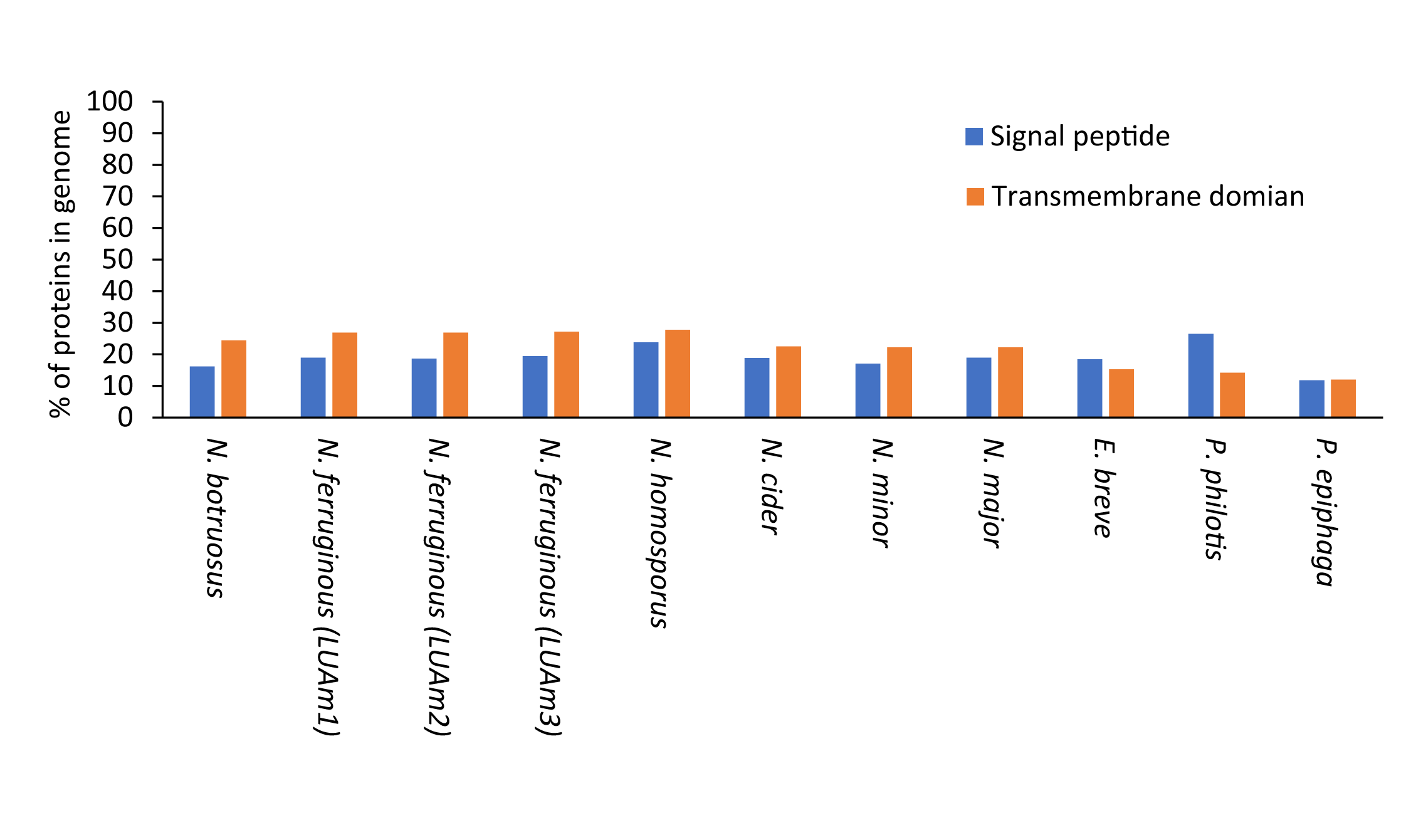

Supplement: S12 Fig — The percentage of proteins in each genome predicted to contain either signal peptides or transmembrane domains is shown. (TIF) [file ppat.1011510.s012.tif]

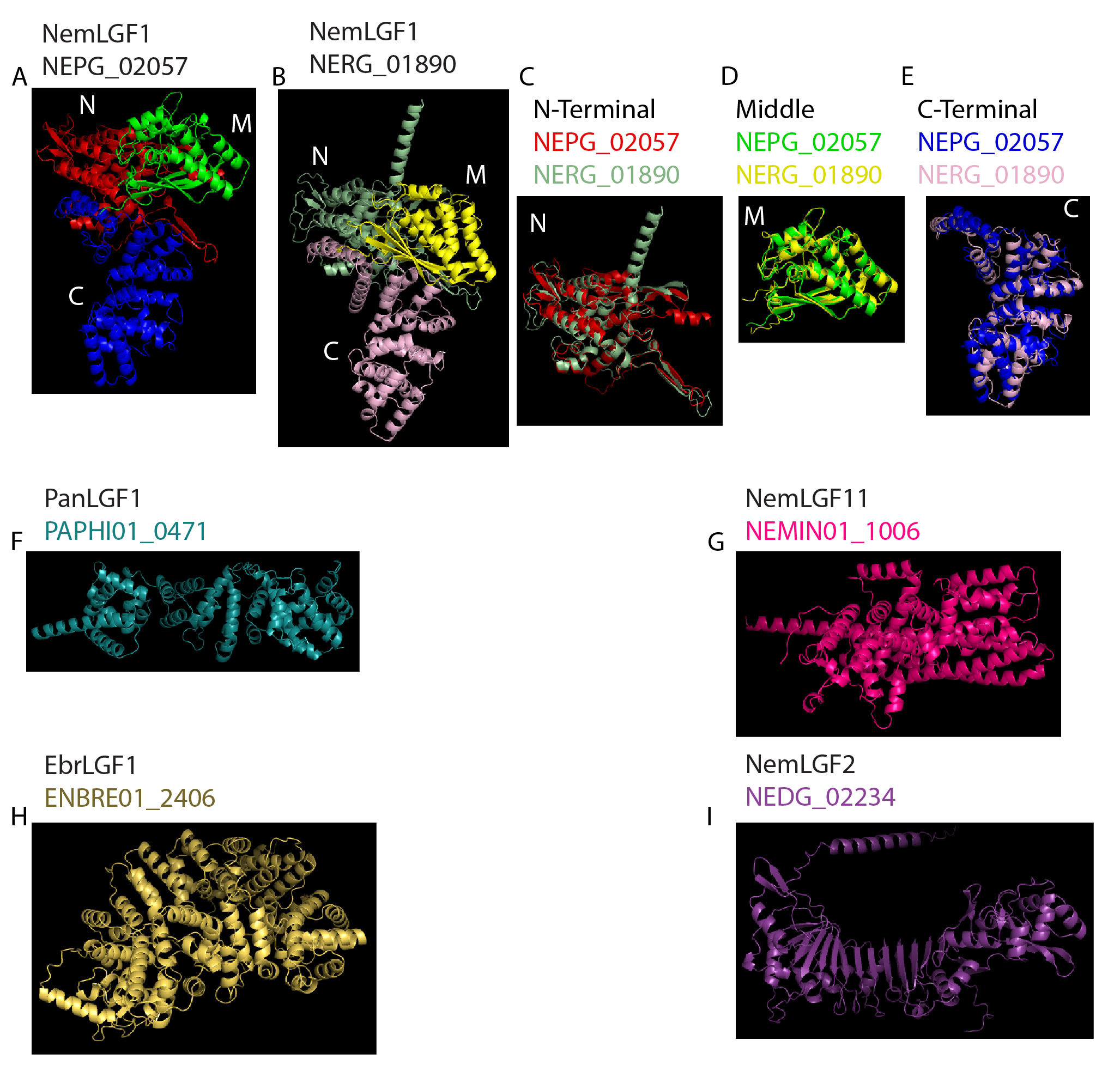

Supplement: S13 Fig — (A-B) AlphaFold models of N. parisii NEPG_02057 (B) and N. ausubeli NERG_01890 (B). (C-E) Aligned structures of the N-terminal (C), middle (D), and C-terminal (E) domains. N, N-terminal. M, middle. C, C-terminal. (F-I) AlphaFold models of PanLGF1 member PAPHI01_0471 (F), NemLGF11 NEMIN01_1006 (G), EbrLGF1 ENBRE01_2406 (H), and NemLGF2 NEDG_02234 (I). (JPG) [file ppat.1011510.s013.jpg]

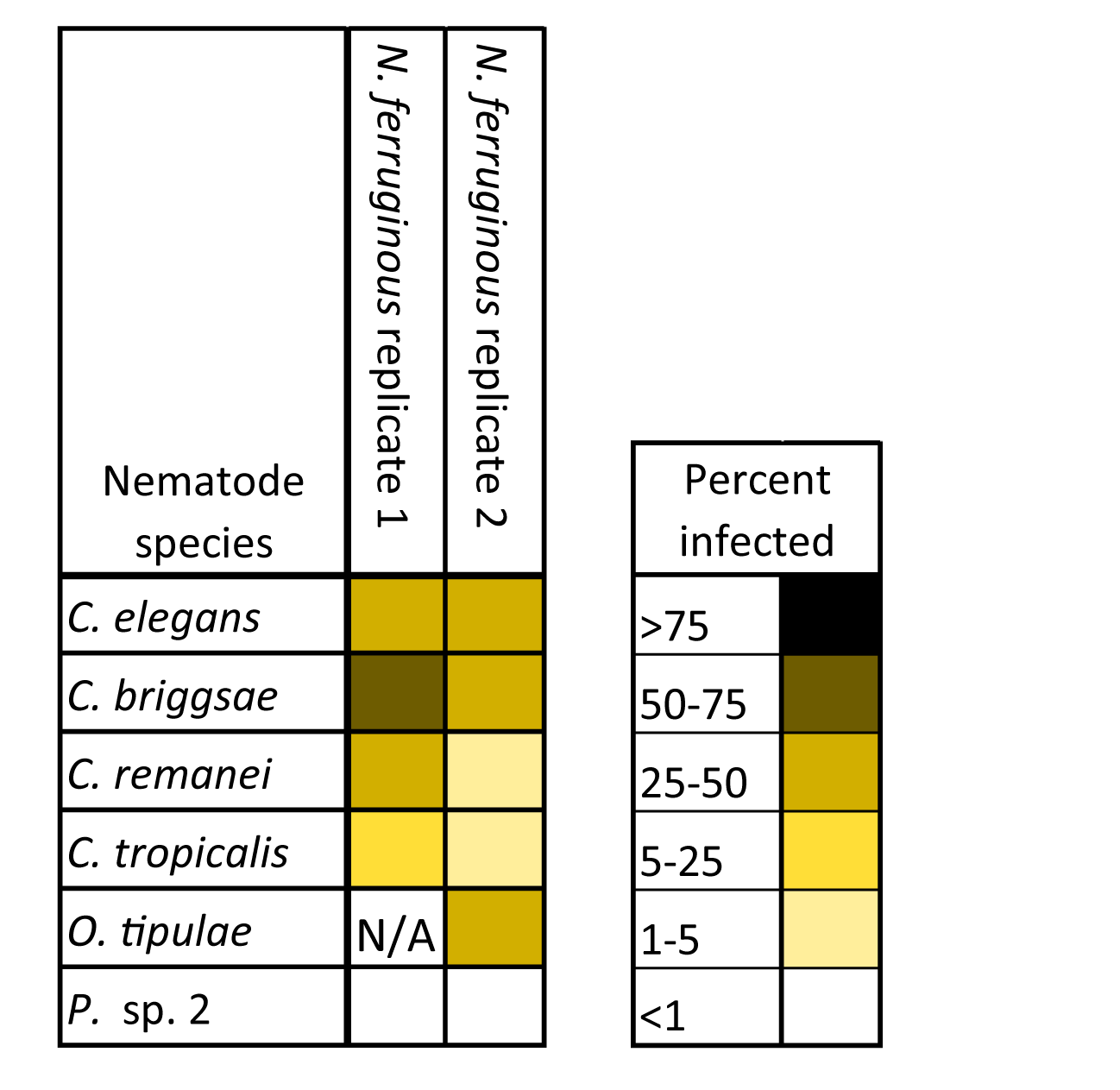

Supplement: S14 Fig — Six species of L1 stage nematodes were infected with either 20 (replicate 1) or 40 million (replicate 2) N. ferruginous (LUAm3) spores. After 96 hours of incubation with spores, animals were fixed and stained with DY96. Percent of each population of animals infected with each species of microsporidia. Data is displayed as a heat map with host species in rows, each N. ferruginous replicate in columns, and the value of each cell being the percent of each population that displayed newly formed microsporidia spores. Legend is displayed at the right. 50–196 animals were counted for each sample. (TIF) [file ppat.1011510.s014.tif]

**A***N. botruosus***B***N. cider***C***N. ferruginous*

Direct Yellow 96

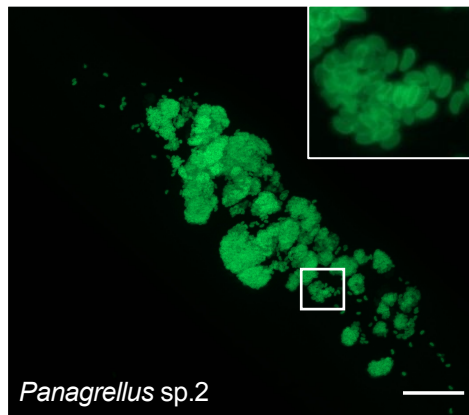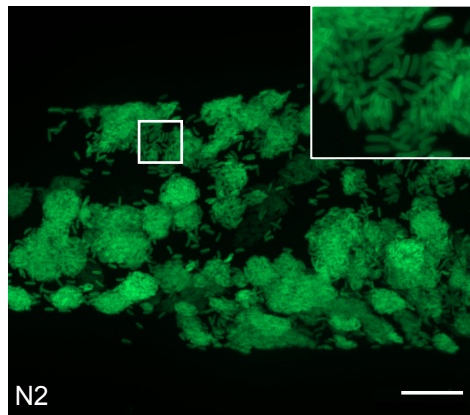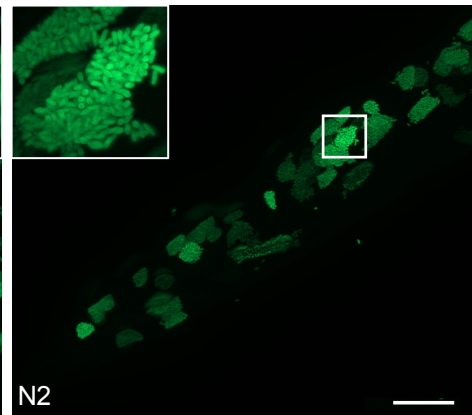**D**

Uninfected

*N. cider**N. ferruginous*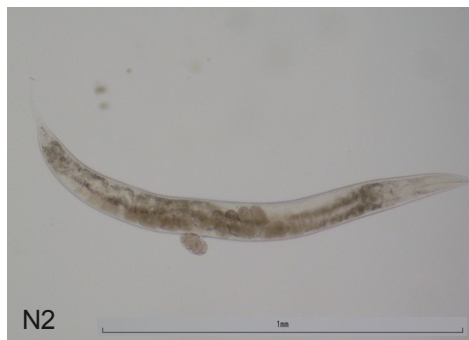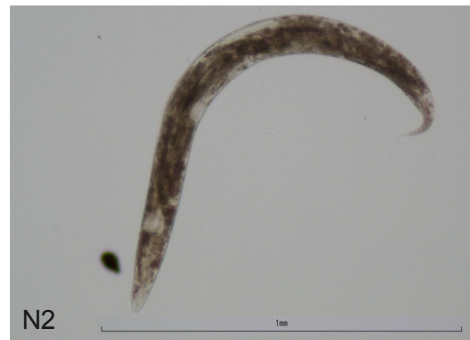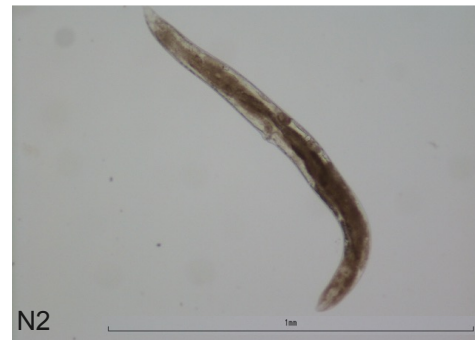

Supplement: S15 Fig — (A-C) L1 stage animals were exposed to spores and incubated for 96 hours, fixed, and stained with DY96. Representative images were taken with the apotome module of a ZEISS Axio Imager at 63x magnification. Multiple z-planes were imaged, and a maximum intensity projection is displayed for each sample. Scale bars, 20 μm. (A) Panagrellus sp. 2 infected with 28 million N. botruosus spores. (B) C. elegans N2 infected with 4 million N. cider spores. (C) C. elegans N2 infected with 40 million N. ferruginous (LUAm3) spores. (D) C. elegans N2 animals were either infected with 3 million N. cider spores or 3 million N. ferruginous spores for 5 days. Images taken with Nikon Eclipse Ni at 10 x magnification. (PDF) [file ppat.1011510.s015.pdf]

*Nematocida cider*

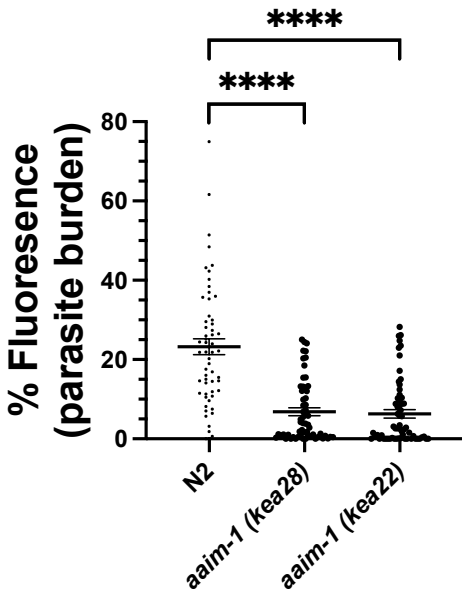

Supplement: S16 Fig — N2 and aaim-1 mutant animals infected with 4 million N. cider spores, fixed at 96 hours, and stained with DY96. 20–30 worms quantified per replicate. The percentage of the animal containing DY96 signal is shown. Mean ± SEM represented by horizontal bars. P-values determined via One-way Anova with post hoc. Significance defined as **** p < 0.0001. (PDF) [file ppat.1011510.s016.pdf]
