# Supplementary material for: Genomic and phenotypic evolution of nematode-infecting microsporidia
Source: PLoS Pathog. 2023 Jul 20;19(7):e1011510. doi: 10.1371/journal.ppat.1011510 (PMC10393165; doi:10.1371/journal.ppat.1011510)
Supplement: Attachment — Submitted filename: plospath_microgenome_response to reviewers_021223_final.pdf [file ppat.1011510.s026.pdf]

We thank the editor and reviewers for their comments. We have provided a revised manuscript (where all changes are highlighted) to address the concerns raised by the reviewers. These changes have been incorporated into the manuscript (including an additional supplemental figure) and we have provided a point-by-point response to each of the reviewer's comments.

Dear Reinke,

Thank you very much for submitting your manuscript "Genomic and phenotypic evolution of nematode-infecting microsporidia" for consideration at PLOS Pathogens. As with all papers reviewed by the journal, your manuscript was reviewed by members of the editorial board and by several independent reviewers. In light of the reviews (below this email), we would like to invite the resubmission of a significantly-revised version that takes into account the reviewers' comments.

This study combines genomic, computational and phentoypic data for 9 species of microsporidia that infect nematodes. Excellent computational analyses are presented and all reviewers agree that this body of work provides a novel resource to facilitate future research on this group of parasitic microsporidia. All reviewers applaud the authors for their context investigating various phenotypic traits that differ among the microsporidia but feel that some of the experimental validation to support the conclusions drawn are underdeveloped, and this certainly needs to be addressed. Reviewer 2 lays out a compelling template how to re-work the manuscript, into a more coherent flow that integrates the various phenotypes to provide a more compelling, evidence-based dataset that supports some of the conclusions made. Alternative explanations should be raised, in particular the support for the Horizontal Gene Transfer of a specific gene family is interesting, but needs to be vetted more appropriately - it may very well be correct, but alternative explanations also exist that are not discussed. The manuscript has the potential to lay the foundation for a new field of study that will be valuable for the community, but the data needs to be more coherently integrated, with supporting evidence, which is the primary challenge to rectify prior to publication.

We cannot make any decision about publication until we have seen the revised manuscript and your response to the reviewers' comments. Your revised manuscript is also likely to be sent to reviewers for further evaluation.

When you are ready to resubmit, please upload the following:

[1] A letter containing a detailed list of your responses to the review comments and a description of the changes you have made in the manuscript. Please note while forming your response, if your article is accepted, you may have the opportunity to make the peer review history publicly available. The record will include editor decision letters (with reviews) and your responses to reviewer comments. If eligible, we will contact you to opt in or out.

[2] Two versions of the revised manuscript: one with either highlights or tracked changes denoting where the text has been changed; the other a clean version (uploaded as the manuscript file).

Important additional instructions are given below your reviewer comments.

Please prepare and submit your revised manuscript within 60 days. If you anticipate any delay, please let us know the expected resubmission date by replying to this email. Please note that

revised manuscripts received after the 60-day due date may require evaluation and peer review similar to newly submitted manuscripts.

Thank you again for your submission. We hope that our editorial process has been constructive so far, and we welcome your feedback at any time. Please don't hesitate to contact us if you have any questions or comments.

Sincerely,

Michael E. Grigg
Guest Editor
PLOS Pathogens

P'ng Loke
Section Editor
PLOS Pathogens

Kasturi Haldar
Editor-in-Chief
PLOS Pathogens
orcid.org/0000-0001-5065-158X

Michael Malim
Editor-in-Chief
PLOS Pathogens
orcid.org/0000-0002-7699-2064

****

This study combines genomic, computational and phentoypic data for 9 species of microsporidia that infect nematodes. Excellent computational analyses are presented and all reviewers agree that this body of work provides a novel resource to facilitate future research on this group of parasitic microsporidia. All reviewers applaud the authors for their context investigating various phenotypic traits that differ among the microsporidia but feel that some of the experimental validation to support the conclusions drawn are underdeveloped, and this certainly needs to be addressed. Reviewer 2 lays out a compelling template how to re-work the manuscript, into a more coherent flow that integrates the various phenotypes to provide a more compelling, evidence-based dataset that supports some of the conclusions made. Alternative explanations should be raised, in particular the support for the Horizontal Gene Transfer of a specific gene family is interesting, but needs to be vetted more appropriately - it may very well be correct, but alternative explanations also exist that are not discussed. The manuscript has the potential to lay the foundation for a new field of study that will be valuable for the community, but the data needs to be more coherently integrated, with supporting evidence, which is the primary challenge to rectify prior to publication.

Reviewer's Responses to Questions

Part I - Summary
Please use this section to discuss strengths/weaknesses of study, novelty/significance, general

execution and scholarship.

Reviewer #1: The paper by Lina Wadi describes the acquisition and analysis of multiple closely nematode-infecting microsporidia strains.

The paper is well written, and the presented analyses seems thoroughly performed.

Reviewer #2: This study reports a combination of genomic and phenotypic data on multiple genera of nematode-infecting microsporidia. It provides a novel resource to enhance future research on this recently discovered group of parasites. The characterization of Nematocida phenotypes in a phylogenetic context makes a particularly valuable contribution – microsporidia are understudied and can be challenging to work with; this study takes advantage of the experimental tractability of nematode-infecting microsporidia to measure multiple traits across the genus.

I reviewed this manuscript with a member of my lab group. We found the methods to be sound and the writing clear. We overall feel positively about the work. Below, we highlight cases where claims require more support and justification from the data and analyses. Several elements of the paper would be strengthened with more biological grounding - the discussion of large gene families for example. Finally, many of the figures are so data-rich that it's challenging to extract the main point.

Reviewer #3: This manuscript reports and analyze genomes of nine species of nematode-infecting microsporidia, including two species in a distinct clade more closely related to human pathogens than the previously studied Nematocida, and three newly described Nematocida species. This adds considerably to the previous four Nematocida species genomes that were available, although the present assemblies are less complete. A particularly intringuing result concerns the apparent horizontal transfer in a gene family between distant genera of nematode-infecting microsporidia. The authors also provide data regarding infection properties and morphology of the three new species.
Overall, this is a useful and extensive characterization of nematode-infecting microsporidia.

Part II – Major Issues: Key Experiments Required for Acceptance
Please use this section to detail the key new experiments or modifications of existing experiments that should be <u>absolutely</u> required to validate study conclusions.

Generally, there should be no more than 3 such required experiments or major modifications for a "Major Revision" recommendation. If more than 3 experiments are necessary to validate the study conclusions, then you are encouraged to recommend "Reject".

Reviewer #1: I found however, the paper to be very descriptive. However, I believe this paper provides some important insight into microsporidian genome evolution. I provide some feedback the authors could use to further improve impact to the entire field, as opposed to center their findings primarily on nematode microsporidians. In particular, it would be interesting to see if the patters the authors observed extrapolate to other microsporidians. In particular, there are

multiple closely related species in the Encephalitozoon/Ordospora clade that have sequences available and could be used to investigate similar patterns at larger scale.

Because the focus of this paper is on nematode-infecting microsporidia we have limited our analysis to these types of microsporidia. However, we have previously looked at large gene families in the Encephalitozoon/Ordospora clade and found similar patterns to what we observe with *Nematocida* (Reinke et al., Nature Communications, 2017). We rewrote an introduction paragraph to include more information on what has previously been reported about these families.

Below are some comments/suggestions.

- L126: Using OrthoFinder29, we generated a phylogenetic tree of our nine newly assembled. Shouldn't this be: using OF we identified Orthologous sequences, which we used to produce phylogenetic trees?

Orthofinder is a program that both identifies the orthologous sequences and can generate a phylogenetic tree. We have rephased this line to make this point clearer.

- L215 : exception of NemLGF2, which shares similarity to Leucine Rich Repeat proteins. The presence and molecular analyses of Leucine Rich Repeats have been published before and should be not considered new. See Campbell et al. 2013 Plos Genetics for a thorough analysis of these proteins.

We are not making a claim that the presence of Leucin Rich Repeat proteins in microsporidia is new, just that these NemLGF2 proteins contain them. We have now rewritten this line and added the Campbell reference to clarify this point.

- L296: We observed that spore size is flexible throughout Nematocida species… What about intra-strain? If the authors want to emphasize size difference among species, one can expect those sizes not to vary within a species. Can the authors show this?

Several strains have been previously measured for two species in Nematocida and the variation reported is less than 10% (Zhang et al, Plos Pathogens, 2016). We have now updated the results in line 340-341 to include this point.

- L3-4. These results provide further support for microsporidian tube length being a factor in determining which tissues are infected.
How about the PTP genes? Do they behave in different ways among species? For example, are these present in different copy numbers etc that could reflect the observed phenotypic variability?
The polar tube proteins are not known in *Nematocida* (Tamim El Jarkass et al, Cellular Microbiology, 2020). We have added to the end of this sentence to indicate this.

- L329: …present in any other microsporidia species, the most likely explanation is that this family was horizontally transferred between a species in each genus…

Or perhaps these appeared de novo?

HGT is testable and if it happened here, one would expect a very high sequence identity in these proteins among the clades. Is this being observed here? Any evidence these genes evolve differently than others?

Although we favor HGT as being the most likely explanation for the origin of the NemLGF1 gene family in the two microsporidia clades, we have now rewritten the discussion to make explore the other possibilities for the origin of this family. We also report the sequence identity between the NemLGF1 proteins which we analyzed in Figure 5. Between *N. parisii* NemLGF1 protein NEPG_02057 and *P. epiphaga* protein PAEPH01_0103, there is 22% sequence identity. Between NEPG_02057 and the NemLGF1 protein NERG_01890, there is 27% identity.

Taken from lines 375-390, this our explanation of the various scenarios that could explain our observation of this protein family:

"Here we show that one of these large gene families, NemLGF1, is present in both *Nematocida* and *Pancytospora* species. There are three possibilities that could explain this observation. One, the protein family in the two groups of species could have evolved *de novo* independently of one another. This type of convergent evolution is rare and given the high predicted structural similarities that we observe, this scenario is unlikely [47]. Two, this protein family could have existed in the last common ancestor of *Nematocida* and other microsporidia, and then was lost in all lineages except for *Pancytospora* and part of *Nematocida*. From the genomes we analyzed, this would mean that this family would have been lost 7-9 times (*Nematocida Displodere* group, *N. minor*, *V. corneae*, *E. breve*, Enterocytozoon, Nosematida, Glugeida, Neopereziida, and Amblyosporida) [33]. Although this explanation is also unlikely, additional sequencing of microsporidia genomes could provide more support for or against this scenario. Three, this family was horizontally transferred between a species in each genus, though which genus was the recipient, and which was the donor, is not clear. Horizontal gene transfer is increasingly being appreciated as having a role in the evolution of eukaryotic microbes and we believe this to be the most likely explanation [48]."

Reviewer #2: 1. Connection between claims and data/analyses: There are results presented in the Abstract as major conclusions that we felt were not sufficiently supported in the main text.

A) The suggestion of horizontal gene transfer between distantly related nematode-infecting microsporidia is a pretty big claim, especially given that there are no prior reports of horizontal transfer between microsporidia. We didn't feel that this was an unreasonable hypothesis given the pattern observed, but we felt the data were insufficient to make horizontal gene transfer the "mostly likely explanation" (line 329) to put forward. We would like see either 1) more justification of this claim (are there other data that support this hypothesis? Including a supplemental phylogeny for other large, distributed gene families so we can make a comparison?) and/or 2) more qualification of this claim, including presentation of alternate hypotheses (undersampling of the microsporidia phylogeny?) and the type of data necessary to test them.

As mentioned above in response to reviewer one, we have now modified the discussion to mention the three possibilities for the origin of the NemLGF1 family including addressing how sequencing more microsporidia species could impact our conclusion.

B) Lines 19-21 argue that these large gene families are likely used to interact with host cells. This idea is raised again in lines 79-80 of the Introduction, though with limited clarification of the prior result in the literature, then again in the Conclusion lines 325-327 stating that "these families were enriched for secretion signals and transmembrane domains." We didn't see this analysis of enrichment in the Results, beyond Figure 4 showing the distribution of signal peptides and transmembrane domains in expanded gene families. What is the distribution of signal peptides and transmembrane domains in a random sampling of gene families that are not expanded? Are they less likely to have these domains than expanded families?

We previously showed that *N. Parisii* proteins that were in contact with the host cytoplasm in *C. elegans* were enriched in large gene families. We also showed that these large gene families were enriched in having either signal peptides or transmembrane domains (Reinke et al., Nature Communications, 2017). We have now added a new figure (Fig. S12) which shows the percent of proteins containing predicted signal peptides or transmembrane domains in our newly sequenced genomes.

2. The large gene families section was also an area where we felt that substantially more discussion and biological grounding was needed for the reader to understand if, and why, they matter. What are the hypotheses for why these gene families might be expanded, particularly when the major trend is gene loss? To what extent are the observed patterns consistent with those hypotheses? How sensitive are these gene family results to the specific parameters used in OrthoMCL? Can the authors justify their choice of parameters as the most appropriate for detecting large gene families in microsporidia?

This analysis was performed as previously described in (Reinke et al., Nature Communications, 2017). Large gene families in other microsporidia have also been reported (Nakjang et al, GBE, 2013.) . We have now modified the introduction to maker clear what is previously known about these families.

We felt similarly about the protein structures – these protein structures can be modeled and presented, but what do we learn from them? We thought these models needed more justification if they were to be included, or alternately could be excluded with no impact on the paper.

We feel that the predicted structures for NemLGF1 proteins provides support that that members of this family from the different clades shared a common last ancestor. We feel that showing high structural similarity provides evidence against these families occurring via convergent evolution. We have now revised our discussion to include this justification.

3. The figures: As noted above, the figures opt to show as much information as possible, which is valuable as a resource, but makes them very hard to look at and interpret.

We understand that this is a balance, but we agree with the reviewer that our paper will be a valuable resource and thus we feel that it is important to include as much information as possible in the figures. We have improved the clarity of the figures as noted in some of the responses below.

Figure 2: can the authors somehow delineate (maybe using boxes) data associated with the nematode-infecting microsporidia? This subset of the data is currently hard to track.

We have now revised this figure by using colored boxes to highlight the nematode-infecting microsporidia.

Figure 3: Same issue.

We have now revised this figure by using colored boxes to highlight the nematode-infecting microsporidia.

Figure 4: this figure would be helped with a more supportive legend that unpacks the significance of the different components of the figure. Alternately, a more valuable figure would be a comparison of signal peptide and transmembrane domains in expanded vs. non-expanded gene families.

We have now modified the figure legend to help improve the understanding of this figure. As described above, we have now added an analysis of the presence of signal peptides and transmembrane domains in our newly sequenced microsporidia species in Figure S12.

Figure 5: this figure is too dense and complex. We thought B-F did not substantially contribute to the paper. For A, is there a way of simplifying these data, and modifying the color scheme, to make the pattern you're most interested in (the potential for HGT) clearer?

For 5A, we agree that showing the evolutionary relationships of ~1000 sequences is difficult, but we do not know of a clearer way to display this data. For figure 5B-F we disagree with the reviewers that this does not add to the paper as explained in our response above.

Figure S1: a table would be better for presentation of these data. The heat map is inappropriate given the scale of the data.

The data is already presented in tabular form with the values shown. We feel that the coloring helps with looking at this figure, but readers are free to disregard the coloring and just focus on the values.

Fig. S4-S11: same suggestion as for Figure 2.

We have now revised these figures by using colored boxes to highlight the nematode-infecting microsporidia.

Fig. S12: this is too small and uses too many colors to be interpretable. We don't feel that it's necessary. If it does make a point that the authors think is critical, can it be simplified to highlight that point?

As this analysis is a negative result and is difficult to visually display, we have now removed this analysis and figure from the manuscript.

Fig. S13: we didn't feel that this added to the paper and the second sentence of the title is an overstatement.

We disagree with the reviewer that this figure on molecular modeling of LGF families doesn't add to the paper. Very little is known about the function of these proteins, and structural modeling provides at least some additional information on these proteins. The second sentence of this figure legend title was added in error, and we have now removed it.

Fig. S14: could this be revised to show the actual data, with error estimates (either binomial if replicates are kept separate or standard error if averaged)? That would be more appropriate and might also help understand if infection rates really declined with increased dose (they appear to, but we don't know the extent to which that arises in part from a wide confidence interval around the estimate).

We feel that showing the infection data as heatmaps is the best way to visualize this data. However, we have now included Supplementary data file S1 that has the raw data for the infection measurements so that the exact values can be looked at and the reproducibility between experiments can be ascertained.

All that being said, we thought Figure 1 was a very nice presentation of data!

We thank the reviewers for their comments.

Reviewer #3: 1) This manuscript describes two new species of microsporidia. Please conform to some minimal standard of species descriptions. Deposit type specimen (live cultures of infected nematodes are fine). Make the basis for raising a new species explicit, for example genome divergence. Latinize the names. Maybe provide a light microscopy picture of spores (either isolated or in the host).

Our descriptions of the three species of microsporidia that we identified include the location that the species was found, the host species, host tissues infected, spore morphology, polar tube length, and assembled genome. This level of description is beyond what is typically included for microsporidia species identification (Murareanu et al, 2021, mBio). We now mention the spores are deposited in our collection and make the basis of them being new species explicit. To the best of our knowledge, the names we chose are latinized.

2) Phylogeny and character mapping: the authors use a parsimony logic to infer character evolution. I would be more prudent in the conclusions as there are few species in Fig. 7. For example, the claim in the abstract that the branch leading to N. homosporus lost the ability to invade multiple tissues is weak and needs to be removed.

We have removed this line from the abstract.

Part III – Minor Issues: Editorial and Data Presentation Modifications
Please use this section for editorial suggestions as well as relatively minor modifications of existing data that would enhance clarity.

Reviewer #1: see some comments above.

Reviewer #2: 64 revise as "suggesting that these traits are quite evolutionarily labile in microsporidia." Plastic has a distinct meaning that isn't what you want here. It would also be helpful to have a little more context with regards to what is meant by "morphological and infection properties"

Changed to suggested phrasing and defined morphological and infection properties.

79-80 provide more context for this prior result, given that it is a major feature of this paper
We have now added another sentence in this section to provide more context for microsporidia large gene families.

84 to be involved

Changed

87 analysis

Changed

98 the results shed limited light on evolutionary mechanisms; a better fit would be: "Our study reveals the evolutionary relationships of nematode-infecting microsporidia and…"

Changed

104 have revealed

Changed

188-190 This sentence collides with the prior sentence (181-182) and subsequent sentence (192-194) indicating that there are several gene families that have more than 10 members present in multiple species. Is this referring to only microsporidia that don't infect nematodes?

Yes, that sentence was referring to microsporidia that don't infect nematodes. We have modified the sentence to make this clear.

296 variable instead of flexible

Changed

299 How were spore clusters measured? What is their biological significance?

Presence of large spore clusters was based on visual inspection of either DY96 stained spores in Figure S15 or from previously published images[19]. We have now add this to the methods

299: variation in polar tube length, rather than flexibility

Changed

306 "Together, our results support evolutionary change in both morphological features and specificity in Nematocida, notably with loss and gain of tissue specificity"

Changed

315-317: can the authors provide an analysis to support this claim? How conserved are metabolic proteins vs. host-interacting proteins?

We mean that these metabolic proteins are present which we show in Figure 2 and 3. This has now been clarified in the discussion.

333 do you mean: "...horizontal transfer of genes between microsporidia species…?"

Changed

338 "Microsporidia have been shown to display substantial variation in their phenotypic traits." (consistent with our prior comments, flexibility and plasticity imply something more than what we think you mean here)

Changed.

471-472 can the authors clarify this sentence "Families not classified as…" – what analysis was being done here?

Only families that contained either signal peptides or transmembrane domains in greater than >50% of proteins were included in our analysis. We have now rewritten this sentence to clarify this point.

497-500 indicate that a wide variety of doses were used across species in the exposure experiments. Is there a rationale for this and how does it affect interpretation of the results?

We chose these doses so that average percentage infection was between 80-99% in either *C. elegans* or the host species the microsporidia was found in, with the exception of *N. ferruginous* where this level of infection was not possible to achieve. We have now added this rational to

the methods and we have also added supplementary data S1 that contains the raw data of these measurements.

649, 664 presence

Changed

Reviewer #3: - The authors write that the nematode-infecting microsporidia Enterospectra and Pancytospora form a sister clade to Enterocytozoon (e.g. on line 92), and that Enterospectra and Pancytospora with Vittaforma corneae form a sister clade to Enterocytozoon, which appears more correct in their analysis (e.g. on lines 136, 160). Such assertion is contingent on the species they use for their analysis, so please make this clear that it is using the set of species shown in Fig. 1.

We have now revised this sentence to improve its clarity.

- Horizontal gene transfer of LGF1 family. In Fig. 4, only nematode-infecting microsporidia plus Vittaforma cornea are represented. Are all of these gene families absent in the other genomes shown in Fig 1?

Yes, except for NemLGF26, as described in the results. We have now added this analysis to the methods section on large gene families.

Please explain how the horizontal transfer is inferred and explicit the scenarios in the section in lines 198-201.

We have now revised this part to make it clearer.

line 200: the support on the tree in Fig S12 is not readable.

As we described in our response above, we have now removed the previous Fig. S12.

- Please indicate the reference for the raw sequence reads in Table S1. The accession number in the Methods (SRX13790799) seems to point only to reads to a Nematocida major archive.

We have now modified the data availability statement to indicate that all the sequence read archive numbers are now in table S1.

lines 17-18: this sentence is not informative: basically any phylogenetic analysis would provide two groups of species. The same applies in other places in the manuscxript.

We disagree with the reviewer that describing *Nematocida* as two groups is not informative. We show throughout the manuscript that these two groups have different genomic and phenotypic properties and thus we feel that classifying them as separate groups facilitates our description of them. We have however rewritten the sentence in the abstract to make it clearer that these groups are based on both genomic and phenotypic properties.

33: give an at least approximate genome size here.

Added.

58-60: within this general paragraph, do you now focus on nematode-infecting microsporidia in this sentence?

No, this paragraph is focused on microsporidia in general and we have yet to introduce nematode infecting microsporidia at this point in the introduction. We have however clarified a related point in the results about when we are refereeing to tissue specificity of microsporidia in general and when we are refereeing to nematode infecting microsporidia in particular.

70-75: the succession of sentences is difficult to follow.

We have now rewritten this section to improve the clarity.

87: 'analysis'

Changed

93: sentence structure to revise, for example add 'that' before 'most'

Changed.

104-107, etc: the logical flow is also difficult to follow here.

This section has been rewritten.

155: revealed

Changed.

184: such AS NemLGF11

Changed.

188-90: convoluted sentence.

Changed.

194: remove one 'this'

Changed

276, 279: do not use the word 'correlate' with a single example.

This correlation is based on over 30 examples as was previously reported (Murareanu et al, mBio, 2021). We mistakenly omitted this reference. We have now added this citation and modified this section to make this point clearer.

286: perhaps replace 'arranged' by 'plotted'.

changed

297: ambiguity that could be resolved by 'both Nematocida groups containing species...'

changed

307: 'throughout speciation' is unclear.

Rephrased

346: a stronger caveat is that this is not strongly supported, even using parsimony.

We feel that this our original caveat on only using a single strain for each species is important and we have kept this sentence in the manuscript.

426, 431: explain why your have to remove ERTm1 contamination in the read data.

Because our laboratory regularly uses ERTm1 for experiments, there exists the possibility of minor contamination of other microsporidia genomes. This explanation has now been added to the methods section.

593: the GPS coordinates seem excessively precise to the nanometer for a millimeter long nematode.

We have now revised these coordinates to avoid being overly precise.

641: Fig 1 legend: explain what the phylogenetic tree is based on.

We have now clarified this in the Figure 1 legend.

649, 664: presence (typo)

Changed

672: change the title: Vittaforma cornea is not a nematode-infecting species.

We have now clarified in this figure legend why *V. cornea* is included.

698: typo PanAgrellus

Changed

705: 'a Nematocida'

Changed

926: rephrase as the L1 larvae do not stay at this stage for 96 hrs.

Rephrased

The respective predicted genome size of the nematode-infecting microsporidia is hard to read on Fig. 1 because of the large variation among all represented microsporidia and is only noted in Fig. 1. Figure S3 could have the species names next to each dot. And/or the genome sizes could be provided in Fig. 7.

The genome sizes have now been added to a revised Figure 7.

Could you explain what Panagrellus "sp. 2" refers to?

Yes, this is the host nematode species that *N. botruosus* was identified in. We have defined this in line 243.

Figure 1: make font sizes larger for the x axis and the legend.

The font sizes for this figure have now been increased.

Figure 3: is this figure important?

This figure more clearly shows conservation of metabolic pathways than Figure 2 does. As reviewer 2 mention a lot of our figures are data rich, so we feal that this point will be easier for readers to understand the way the data is displayed in figure 3.

Figure 5: typo at 'epiphaga'

Changed

Figure 6C: explain what is to be seen in panel C. For example, for the species on the left, does it infect the intestine (this seems contradictory to panel D, is it a rare event?)? In the muscle panels, the staining of microsporidia does not seem to match that of the muscles.

The image of *N. ferruginous* in the intestinal reporter strain shows that this parasite is not infecting the intestine as the parasite fluorescence does not overlap with the GFP fluorescence. For the muscle reporter, we have now revised this figure to include images with increased brightness that more clearly show that there is overlap between the microsporidia fluorescence and the GFP fluorescence.

PLOS authors have the option to publish the peer review history of their article (what does this mean?). If published, this will include your full peer review and any attached files.

If you choose "no", your identity will remain anonymous but your review may still be made public.

Do you want your identity to be public for this peer review? For information about this choice, including consent withdrawal, please see our Privacy Policy.

Reviewer #1: No

Reviewer #2: No

Reviewer #3: No

Figure Files:

While revising your submission, please upload your figure files to the Preflight Analysis and Conversion Engine (PACE) digital diagnostic tool, https://pacev2.apexcovantage.com. PACE helps ensure that figures meet PLOS requirements. To use PACE, you must first register as a user. Then, login and navigate to the UPLOAD tab, where you will find detailed instructions on how to use the tool. If you encounter any issues or have any questions when using PACE, please email us at figures@plos.org.

Data Requirements:

Please note that, as a condition of publication, PLOS' data policy requires that you make available all data used to draw the conclusions outlined in your manuscript. Data must be deposited in an appropriate repository, included within the body of the manuscript, or uploaded as supporting information. This includes all numerical values that were used to generate graphs, histograms etc.. For an example see here on PLOS Biology: http://www.plosbiology.org/article/info%3Adoi%2F10.1371%2Fjournal.pbio.1001908#s5.

Reproducibility:

To enhance the reproducibility of your results, we recommend that you deposit your laboratory protocols in protocols.io, where a protocol can be assigned its own identifier (DOI) such that it can be cited independently in the future. Additionally, PLOS ONE offers an option to publish peer-reviewed clinical study protocols. Read more information on sharing protocols at https://plos.org/protocols?utm_medium=editorial-email&utm_source=authorletters&utm_campaign=protocols
