# Supplementary material for: Genomic and phenotypic evolution of nematode-infecting microsporidia
Source: PLoS Pathog. 2023 Jul 20;19(7):e1011510. doi: 10.1371/journal.ppat.1011510 (PMC10393165; doi:10.1371/journal.ppat.1011510)
Supplement: Attachment — Submitted filename: plospath_microgenome_response to reviewers_052523.pdf [file ppat.1011510.s027.pdf]

We thank the editor and reviewers for their comments. We have provided a revised manuscript (where all changes are highlighted) to address the concerns raised by the reviewers. These changes have been incorporated into the manuscript (including a revised supplemental figure) and we have provided a point-by-point response to each of the reviewer's comments.

Dear Reinke,

The revised manuscript is much improved, and more coherently integrates the datasets. The added alternatives to the HGT hypothesis are welcomed, which was considered the major flaw in the prior version of the manuscript. One of the reviewers felt that some further clarification is necessary, and they provide detailed suggestions how best to address their reservations in text. In conclusion, the authors have generated a broad analysis of microsporidia paired with phenotyping of multiple closely-related and experimentally tractable species infecting nematodes. This paper is poised to become a valuable resource for the community and a source of interesting hypotheses for the field. Please specifically address the concerns raised by Reviewer 2 in the next iteration.

Reviewer's Responses to Questions

Part I - Summary
Please use this section to discuss strengths/weaknesses of study, novelty/significance, general execution and scholarship.

Reviewer #1: (No Response)

Reviewer #2: I re-reviewed this manuscript with a member of my lab group. The manuscript serves as a resource for the microsporidia and a source of hypotheses for the field. The presentation of the manuscript is improved, as is the presentation of the HGT hypothesis. However, we continue to have reservations about the presentation of this idea and the lack of supporting data. We detail these below.

Part II – Major Issues: Key Experiments Required for Acceptance
Please use this section to detail the key new experiments or modifications of existing experiments that should be absolutely required to validate study conclusions.

Generally, there should be no more than 3 such required experiments or major modifications for a "Major Revision" recommendation. If more than 3 experiments are necessary to validate the study conclusions, then you are encouraged to recommend "Reject".

Reviewer #1: (No Response)

Reviewer #2: We appreciate the authors' inclusion of a nuanced discussion of the different hypotheses to explain the presence of the NemLGF1 family in two distantly related genera. A few further modifications:

1.There are sentences in the abstract (line 41) and results (line 230) that present the HGT

hypothesis as the explanation for this result. We agree that HGT is a viable hypothesis to explain the observed pattern. However, the manuscript does not include data to support this hypothesis. Speculation about the HGT hypothesis should therefore be restricted to the Discussion section.

We disagree with the reviewer that our manuscript does not provide strong evidence that HGT is the most likely hypothesis, however we have made the request to restrict our arguments about this hypothesis to the discussion.

2.Including the sequence identities is a good idea (lines 239-242), but in the absence of comparative data, these are not useful as data to support (or not) the HGT hypothesis. We need to know the expected sequence identity for proteins drawn at random from Nematocida and Pancytospora. As Reviewer 1 suggests, if the sequence identity for NemLGF1 members is higher than we'd expect given the genome wide averages, that would provide at least some quantitative support for the HGT hypothesis.

We disagree that calculating the sequence identities between genomes in the two genera would be informative as these sequence identities are typically calculated on alignable residues, which makes the minimum identity typically 20% or greater. Regardless of what the origin was for the last common ancestor of the NemLGF1 proteins in Nematocida and Pancytospora, there has been clear diversification of sequences through speciation and duplication. Thus, we don't think that comparing the identity of NemLGF1 proteins to the average proteome identity provides support either for or against the HGT hypothesis. Using BLAST E-values between recipient and donor taxa has been used to define HGT (Rancurel et al Genes 2017), but we are not aware of any papers on horizontal gene transfer where the requested calculation has been used to provide support for HGT.

3.In lines 239-242 and 363-364, the authors make the point that the high structural similarity of the NemLGF1 proteins in spite of low sequence identities argues for HGT and against convergence. Frankly, this pattern sounds a bit like convergence – more similarity of properties related to function than you might expect given low underlying relatedness. We do agree with the authors' intuition that convergence is an unlikely explanation, but we felt this claim about structural similarity did not help the case.

See below for further explanation of how structural similarity can be used to argue against the possibility of convergent evolution.

4.Following on this point, the authors argue that the NemLGF1 protein structures are highly similar, but we found this difficult to see in Fig. 5 – there's some bits that seem to overlap, some that don't. Is there a statistical framework in which to evaluate structural similarity of these predicted structures? This would help make the point. Moreover, we would benefit from an explanation in the text of how to interpret RSDM values for this purpose and what these values signify in the context of structural similarity.

To provide a statistical framework for interpreting the predicted structures, we searched the predicted structures of NemLGF1 proteins using Foldseek. Searching PAEPH01_0103 gave a top E-value of 1.48e-28 to an *N. parisii* LGF1 protein and no E-values less than 10 for proteins in any other microsporidia besides *Nematocida*.

We expand upon our previous discussion of how structural similarity argues against convergent evolution. Although convergent evolution of entire proteins is thought to be rare, there are example of enzymes where this has been proposed (Galperin, PNAS, 1998) In 16 out of 18 examples of convergent enzymes the structural fold is not shared between the proteins. In the other two cases the RMSD values were 3.4 and 4.8 angstroms. As our values (1.6 Å for N-terminal domain and 1.2 Å for middle domain) are less that, this provides support that NemLGF1 proteins are unlikely to have arisen through convergent evolution. We have modified lines 366-370 of the discussion and included this reference.

Part III – Minor Issues: Editorial and Data Presentation Modifications
Please use this section for editorial suggestions as well as relatively minor modifications of existing data that would enhance clarity.

Reviewer #1: (No Response)

Reviewer #2: 89-91 revise for parallelism

Revised

100-102 with new additions to this paragraph, this sentence can be cut

This sentence has been removed

378 "than" in place of "that"

Revised

400 perturbing
Revised

470 The authors first mention ERTM1 here when talking about removing contaminants. However, ERTM1 is not described as N. parisii until line 614.
We have now modified this line to refer to ERTm1 as N. parisii.

489 Are the methods to predict proteins for M. incurvata and E. canceri the same as in 478?
Yes, and we have now revised the methods to indicate this.

515-516 does this mean that, in Fig. 4, families were marked as green or yellow only if >50% of proteins had a signal peptide or transmembrane domains? This point is critical to interpretation and wasn't clear to us initially. Given that the methods come after the results, this methodological detail should be included in the results and figure 4 legend

Yes, this is how we define the large gene families and we have now updated the results and figure legend to state this.

Figure S1: can the authors add a matrix presenting similarity across species as well as this between-species matrix?

We have now revised this figure to contain the average nucleotide identity between nematode infecting microsporidia. The identity between strains is greater than 99%, and the identity between species is less than 95%. This information has now been included in lines 137-139.
